# Human Papillomavirus Prevalence and Associated Factors in Indigenous Women in Ecuador: A Cross-Sectional Analytical Study

**DOI:** 10.3390/idr15030027

**Published:** 2023-05-18

**Authors:** José Ortiz Segarra, Bernardo Vega Crespo, Alfredo Campoverde Cisneros, Katherine Salazar Torres, Dayanara Delgado López, Stalin Ortiz

**Affiliations:** 1 Facultad de Ciencias Médicas, Universidad de Cuenca, Cuenca 010203, Ecuador; bernardo.vegac@ucuenca.edu.ec (B.V.C.); alfredo.campoverde@ucuenca.edu.ec (A.C.C.); daya-delgado@hotmail.com (D.D.L.); jose.ortizm@ucuenca.edu.ec (S.O.); 2 Instituto Nacional de Investigación en Salud Pública Dr. Leopoldo Izquieta Pérez (INSPI-LIP), Cuenca 010104, Ecuador; 3 Carrera de Medicina, Universidad Católica de Cuenca, Cuenca 010101, Ecuador; zsalazart@ucacue.edu.ec

**Keywords:** human papillomavirus, indigenous women, sexual health, reproductive health, STIs, Ecuador

## Abstract

Cervical cancer (CC) is the second leading cause of death from malignancy in women in Ecuador. Human papillomavirus (HPV) is the main causative agent of CC. Although several studies have been conducted on HPV detection in Ecuador, there are limited data on indigenous women. This cross-sectional study aimed to analyze the prevalence of HPV and associated factors in women from the indigenous communities of Quilloac, Saraguro and Sevilla Don Bosco. The study included 396 sexually active women belonging to the aforementioned ethnicities. A validated questionnaire was used to collect socio-demographic data, and real-time Polymerase Chain Reaction (PCR) tests were used to detect HPV and other sexually transmitted infections (STIs). These communities are located in the southern region of Ecuador and face geographical and cultural barriers to accessing health services. The results showed that 28.35% of women tested positive for both types of HPV, 23.48% for high-risk (HR) HPV, and 10.35% for low-risk (LR) HPV. Statistically significant associations were found between HR HPV and having more than three sexual partners (OR 1.99, CI 1.03–3.85) and Chlamydia trachomatis infection (OR 2.54, CI 1.08–5.99). This study suggests that HPV infection and other sexually transmitted pathogens are common among indigenous women, highlighting the need for control measures and timely diagnosis in this population.

## 1. Introduction

Cervical cancer (CC) is the fourth most common type of cancer and cause of death in women worldwide, with an estimated 604,000 new cases and 341,000 deaths in 2020 [1]. In Ecuador, there were more than 1500 new cases and 813 deaths reported in the same year [1], making it the leading cause of death due to malignant neoplasia, with an age-adjusted rate of 14 × 100,000. Deaths due to this disease are considerably higher in low- and middle-income countries (18.8 × 100,000) compared to high-income countries (11.3 × 100,000) [2].

Human papillomavirus (HPV) is the main causative agent of CC and the most widespread sexually transmitted infection worldwide [3]. There are over 100 HPV genotypes, 40 of which exclusively infect the cervical mucosa [4]. Based on their oncogenic potential, they have been classified into high-risk (HR) types, including 16, 18, 31, 33, 35, 39, 45, 51, 52, 53, 56, 58, 59, 66 and 68, associated with invasive cancer and lesions; low risk (LR) types, such as 6, 11, 42, 43 and 44, causing genital warts; and types 26, 34, 40, 54, 55, 57, 61, 67, 69, 70, 71, 72, 73, 82, 83 and 84 of unclassified risk [5,6,7]. HPV DNA has been detected in 99.7% of women with CC [8,9], with the most prevalent genotypes being 16, 18, 31, 33, 35, 45, 52 and 58 [10,11].

To develop CC, a prior HPV infection is necessary, but the presence of the virus does not always lead to disease. Enhanced risk of developing CC is associated with both host- and virus-related factors. Host factors include age, number of sexual partners, age of sexual debut, number of births and abortions, prolonged use of oral contraceptives, smoking, and genetic factors [8,12]. The virus-related risk factors are viral load, multiple infections, or co-infections with other viruses (such as herpes viruses and HIV) [13,14,15].

In addition, co-infection with other pathogens, such as C. trachomatis, Gardnerella, Candida, Trichomonas, Mycoplasma hominis, Ureaplasma urealyticum and Treponema pallidum, is associated with inflammatory processes of the cervix, which could facilitate the entry of HPV and increase the risk of HPV-induced disease [16,17].

In Ecuador, the National Institute of Statistics and Censuses (INEC) characterize indigenous women based on self-identification and belonging to an ethnic group. Of the 1,018,176 people who define themselves as indigenous in Ecuador, 50.9% are women, and 49.1% are men [18]. They are grouped into 14 ethnic groups [19], with women from the Kichwa and Shuar ethnic groups accounting for 95% of the indigenous population. 

In indigenous women, social and economic determinants, such as lack of access to health information, prolonged time to the diagnosis of sexually transmitted infections (STIs), and discriminatory practices, are some of the important factors that may contribute to the development of CC. Other factors include early marriages, having multiple sexual partners, and no use of condoms as a barrier method [20].

In previous studies, the established prevalence of HPV in Latin American countries varied. In Cuenca, Ecuador, it was 50.3% [21]; in Peru, 50.6% [22]; in Quito, Ecuador, 49% [23]; and in Chile, 29.2% [24]. This variation could be related to both the age of women and the different methods used for diagnosis in these studies. Likewise, in the indigenous populations of South America, the rates of HPV infection differ from 5.9% in Bolivia [25] to 46.7% in Argentina [26].

There are several studies on the detection of HPV in Ecuador, most of which are performed in women with cervical lesions treated in hospital settings [23,27,28,29] and others in the general population. However, there is not enough information about the HPV status in indigenous populations [21,30]. The aim of this study was to determine the prevalence of HPV infection and associated factors in women from the indigenous communities of Quilloac, Saraguro and Sevilla Don Bosco.

## 2. Materials and Methods

### 2.1. Ethical Statement 

The research project was developed in accordance with the principles of the Declaration of Helsinki (adopted by the 64th General Assembly, Fortaleza, Brazil, October 2013) and according to the county laws and regulations that ensure individual protection. The study protocol was approved by the Bioethics Committee of the Central University of Ecuador (approval number COBI EPI DNPI 12 575412) after being selected in the XIII competition organized by the Research Department of the University of Cuenca. 

Prior to the study procedures, all women provided written informed consent in Spanish, Kichwa or Shuar. For participants under 18 years of age, informed consent was obtained from a parent or legal representative. Positive results for STIs and cervical lesions were communicated to the corresponding health centers in the participating communities and affected women received treatment according to the guidelines and protocols of the Ministry of Public Health (MSP). Women with positive HPV cases with negative cytology were scheduled for follow-up cytological control after one year, and women who tested positive for both HPV and cytology were referred for colposcopy examination.

### 2.2. Study Population 

A cross-sectional study was conducted among 396 indigenous women belonging to the Kichwa ethnic groups from the communities of Quilloac and Saraguro in the provinces of Cañar and Loja, respectively, and the Shuar ethnic group from the community of Sevilla Don Bosco in the province of Morona Santiago. These provinces are located in the southern region of Ecuador, where the majority of the population considers themselves indigenous. 

The inclusion criteria included being sexually active, not pregnant, without uterine lesions, and not having undergone medical or surgical treatment for cancer during the study period. Due to geographical and cultural barriers, indigenous women in Ecuador do not have easy access to free services, including the Papanicolaou (Pap) smear provided by the MSP. To reach these women, the research team, together with the health authorities of zones 6 and 7, facilitated several activities. Zone 6 covers the entire population of Azuay, Cañar and Morona Santiago, while zone 7 includes the population of Loja, El Oro and Zamora Chinchipe. 

First, community leaders were contacted to explain the objectives of the research and obtain their approval to conduct the study in their communities. Second, women were invited to the informative sessions by the health center director to inform them about the objectives, potential benefits, conditions and requirements needed to participate in this study. Finally, after providing training to health workers, cervical samples and socio-demographic data were obtained from women who had previously agreed to participate by signing an informed consent form.

### 2.3. Sampling Collection 

The questionnaire was developed by the research team and validated in indigenous communities with similar characteristics, using the split-halves method, which obtained a Spearman-Brown reliability value of 0.83.

The study material was collected using endocervical brushes by the medical doctors from the local community clinics and project investigators. One sample was used for the molecular detection of HPV and STI pathogens, and another for cervical cytology screening. The biological samples were transported and stored properly until processing at the Laboratory of Molecular Biology, Faculty of Medical Sciences, University of Cuenca. The commercial solution used to preserve the DNA was Sure Path (Becton Dickinson, Sparks, MD, USA) which was immersed in the vial used to collect the sample. This solution allowed the sample to be stored at room temperature for 2–3 weeks, and when refrigerated at 4 °C, the sample could be preserved for up to 1 month. 

### 2.4. HPV Genotyping 

For the diagnosis of the 28 HPV genotypes, including 9 low-risk (6, 11, 40, 42, 43, 44, 54, 61, 70) and 19 high-risk or potentially high-risk (16,18,26,31,33,35, 39, 45, 51, 52, 53, 56, 58, 59, 66, 68, 69, 73, 82), the Anyplex II Detection HPV 28 kit was used [31]. This kit is based on Tagging Oligonucleotide Cleavage and Extension (TOCE^TM^), which enables detection of multiple pathogens in a single fluorescence channel of real-time PCR equipment (thermocycler), allowing simultaneous amplification, detection and differentiation of target nucleic acids from various HPV genotypes mentioned above. An internal control (IC) was applied to verify nucleic acid isolation and to detect possible PCR inhibition. The IC is co-amplified with the target nucleic acids within the biological samples. The kit utilizes the human beta-globin genes as an internal endogenous control that ensures DNA purification and verifies the PCR reaction. The uracil-DNA glycosylase system, included in the kit, prevents uracil deletion mutagenesis of DNA molecules by cleaving the N-glycosyl linkage and initiating the cleavage base repair pathway (BER), which controls cross-contamination of samples with amplicons [31].

The detection of seven STI pathogens (Chlamydia trachomatis, Neisseria gonorrhoeae, Trichomonas vaginalis, Mycoplasma hominis, Mycoplasma genitalium, Ureaplasma urealyticum and Ureaplasma parvum) was performed using the Anyplex ^TM^ II STI-7 detection test [32]. 

### 2.5. Data Analysis 

The sample size calculation to determine the prevalence of HPV was performed using an estimated prevalence of 30% [21,30], with a 95% confidence interval and 5% accuracy, in a total population of indigenous women of Kichwa and Shuar (970,321). The required sample size was 323 women. Considering that the different ethnic groups selected for this study had common characteristics, such as a high fertility rate (5.3 children per woman) and a low level of education (with a mean of 6.6 years of schooling), a single calculation of sample size including women of different ethnicities was made [33].

Descriptive and analytical statistics were used in statistical analysis. The association between percentages was evaluated by logistic regression analysis using the Wald statistic. Odds Ratio with a 95% confidence interval (OR, 95% CI) and chi-square confidence interval were considered using the SPSS V20 program. For all data analysis, values of OR > 1 with the lower bound of 95% CI > 1 were considered statistically significant for risk factors, values of OR < 1 with 95% CI < 1 for protective factors, and a p-value of <0.05 for chi-square was considered significant.

## 3. Results

**Figure 1:**
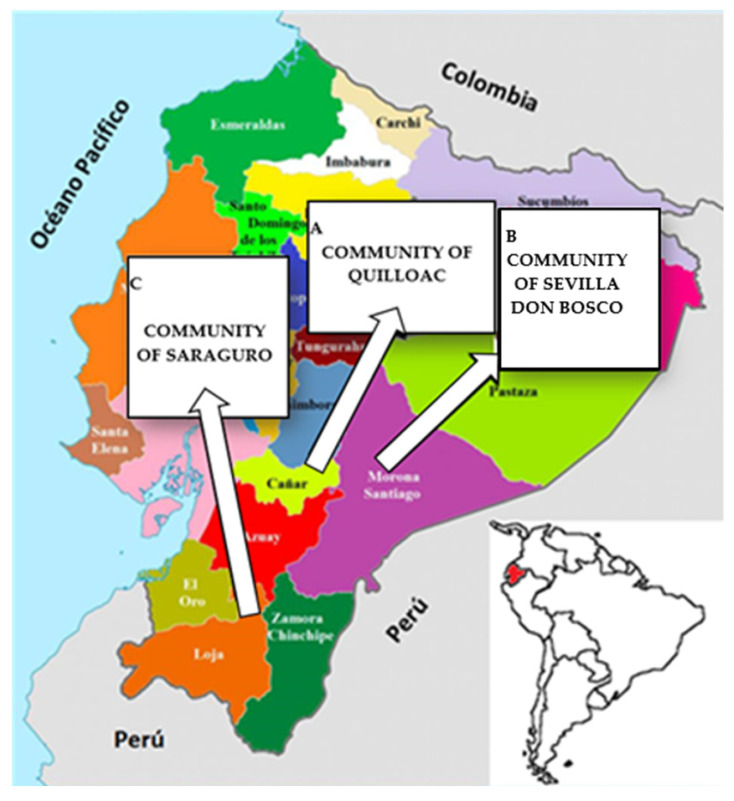
Map of the provinces where the participating communities are located. A: Cañar: community of Quilloac. B: Morona Santiago: community of Sevilla Don Bosco. C: Loja: Community of Saraguro.

Of the 396 women who participated in this study, 131 belonged to the Quilloac community of the Kichwa ethnic group, 145 to the Don Bosco community of the Shuar ethnic group, and 120 to the community of Saraguro of the same ethnicity. Figure 1 shows the map of the provinces of Cañar, Morona Santiago and Loja, where the communities are located. 

**Figure 2:**
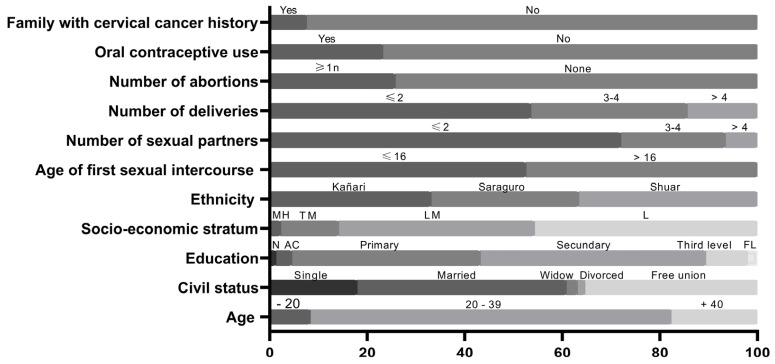
Socio-demographic, sexual and reproductive health characteristics of indigenous women. MH: medium high. TM: typical medium. LM: low medium. L: low. N: none. AC: alphabetization center. FL: fourth level.

The demographic characteristics, as well as the sexual and reproductive history of women enrolled in the study, are presented in Figure 2. The median age was 31 years (interquartile range, 24–37), and 49% (195/396 women) were younger than 31. Most women were in a stable relationship, whether married or living in a free union. A total of 95% of the participants had some level of formal education, 3.28% had studied in alphabetization centers, and only 1.26% were illiterate. The median age of first intercourse was 16 years; 52.5% of women had their first intercourse before the age of 17. Seven out of ten women had up to two lifetime sexual partners, 21.4% between three and four, and 6.5% more than four. Most women belonged to the low socio-economic stratum.

The average number of births was twenty-five percent of women reported having had one or more abortions. In a similar proportion, women reported using hormonal contraceptives. One in 13 women reported that a family member died of cervical cancer.

Table 1 shows the frequency of HPV genotypes in indigenous women according to ethnicity. In the Kañari community, the prevalent HR HPV genotypes were 58, 59, 53 and 31 with 6.87%, 3.05%, 2.29% and 1.53% frequency, respectively. LR HPV genotype six was also found with a 2.29%. The most prevalent HR HPV genotypes in the Shuar ethnicity were 59 with 6.21% frequency, 39 and 58 with 4.83%, and 31 with 4.14%. LR HPV genotype 42 with 6.21%. Finally, the most prevalent HR HPV genotype among the Saraguro community was 39 with 5% frequency; followed by 16, 35 and 68 with 4.17%; and genotype 59 with 3.33%.

Table 2 shows the frequency of HPV, HR HPV and LR HPV in women according to their ethnicity. The frequency of any type of HPV in Kañari, Shuar and Saraguro women was 19.08%, 40.00% and 24.17%, respectively. The frequency of HR HPV in the Kañari community was 14.50%, while LR HPV was 6.87%. In contrast, the percentage of HR HPV was 32.41% and 22.50% in Shuar and Saraguro women, respectively. The percentage of LR HPV was 16.55% for Shuar women and 10.35% for the Saraguro ethnicity. The total frequency for all three communities was 28.28% for any HPV, 23.48% for HR HPV, and 10.35% for LR HPV.

Furthermore, multiple infections were detected in 9.34% of patients, with genotypes 58 and 59 being the most frequent association. The Shuar ethnicity had a higher percentage (12.41%) of multiple infections compared to Saraguro (8.33%) and Kañari (6.87%). A total of 3% of all participants tested positive for three or more subtypes. One woman presented seven genotypes (39, 52, 53, 59, 66, 69, and 42), and two women presented five, three with four, and six with three genotypes. 

Table 3 shows the association between cytological findings and HR HPV. HR HPV infection is significantly linked with the presence of atypical squamous cells of undetermined significance (ASC-US) (OR 4.29; CI 2.01–9.17), low-grade intraepithelial lesions (LSIL) (OR 5.96; CI 2.11–16.89), high-grade intraepithelial lesions (HSIL) (OR 17.16; CI 1.98–148.81), any type of low and high-grade intraepithelial lesion (OR 5.80; CI 3.16–10.65), and even inflammation of the cervix (OR 4.69; CI 1.42–15.53). The association between HR HPV and the severity of the lesion is directly proportional, with a stronger association seen in more severe lesions. 

Table 4 shows the distribution of infection by the seven STI pathogens and HPV in relation to age. A higher frequency of any STI pathogen was diagnosed in women younger than 20 years of age (93.9%, CI 85.8–100%) compared to those in the 20–39 age range (81.2%; CI 76.8–85.7%) and those in the group aged 40 years and over (64.3%, CI 53–75.5%). Similarly, the percentage of HPV infection decreases with age. Thus, in the first age group, HR HPV was found in 39.4% (CI 22.7–56%) and HPV in 15.1% (CI 2.9–27.4%). In the age group of 20 to 39 years, 23.9% (CI 19–28.8%) had an infection with HR HPV, and 10.9% (CI 7.3–14.5%) with LR HPV. As for women aged 40 years and older, 14.3% (CI 6.1–22.5%) were infected with HR HPV and 5.7% (CI 0.3–11.1%) with LR HPV.

Table 5 shows the distribution of socio-demographic, sexual and reproductive health characteristics, genital infections, and inflammation according to the results of HPV and HR HPV. Based on unadjusted bivariate analysis, a significant association was found between ages younger than 19 years (OR 2.29, CI 1.11–4.71), one or more abortions (OR 1.66, CI 1.03–2.69), more than three sexual partners (OR 2.03, CI 1.09–3.78), family history of CC (OR 2.39, CI 1.13–5.08), Chlamydia trachomatis infection 3.91, CI 1.68–9.1), Mycoplasma hominis (OR 1.91, CI 1.19–3.04) and Ureaplasma parvum (OR 1.64, CI 1.04–2.59). Although the presence of inflammation of the cervix was associated, it was not statistically significant (OR 3.05, CI 1.75–7.97). For HR HPV, the factors significantly associated were age younger than 19 years (OR 2.30, CI 1.09–4.82), single unmarried state (OR 1.89, CI 1.08–3.13), more than three sexual partners (OR 1.98, CI 1.04–3.77), Chlamydia trachomatis infection (OR 2.99, CI 1.29–6.92) and Mycoplasma hominis (OR 1, 85, CI 1.13–3.02).

Table 6 shows that, after binary logistic regression analysis, there was a significant improvement in predicting the probability of HR HPV infection (chi-square 17.93, Df 4, *p* < 0.001), achieving a 76.9% probability of diagnosing HR HPV infection when women are 19 years old or younger, have had more than three sexual partners, and have co-infections with Chlamydia trachomatis and Mycoplasma hominis. Furthermore, the logistic regression analysis of risk factors reveals that the variables significantly associated with HR HPV were having more than three sexual partners (OR 1.99, CI 1.03–3.85) and Chlamydia trachomatis infection (OR 2.54, CI 1.08–5.99).

## 4. Discussion

This study describes the prevalence of HPV and other STI pathogens in indigenous women in Ecuador. There is a lack of research on HPV in indigenous women in Ecuador [34]. This study focuses on the communities of Quilloac, Saraguro and Sevilla Don Bosco, where various factors such as socio-demographic, clinical characteristics, and epidemiological data were analyzed to better understand the prevalence and impact of HPV infection. In the literature, a prevalence of 25.6% for any HPV, where 20.40% accounts for HR HPV and 20% for LR HPV, was previously reported in one study of mestizo women in the city of Cuenca-Ecuador [21].

In the current study, an infection rate of 28.28% was detected for any type of HPV, 23.48% for HR HPV, and 10.35% for LR HPV. These results are similar to those found in Paraná-Brazil (28.6% for HR HPV, 19.3% for any HPV) [35] and Paraguay (23.2% for any HPV, 16.1% for HR HPV) [36], but lower than those reported in Colombia (49.2% for any HPV) [37], Brazil (45.9% for any HPV and 34.1% for HR HPV) [35] and Argentina (46.7% for any HPV, 31.3% for HR HPV and 9.8% for LR HPV) [26]. These differences may be attributed to the socio-demographic characteristics, sexual behaviors, and reproductive history of the participants, among other factors that could influence the risk of acquiring viral infection in these populations.

The indigenous women participating in this research had a mean age of 31 years, with an average of three pregnancies, two sexual partners, and their first sexual intercourse at 17 years of age. By contrast, the women included in the Deluca study [26] had a mean age of 30 years and sexual initiation of around 15 years. As is well known, HPV infection is age-dependent, being more common in women older than 25 years [38]. This may explain, in part, the lower prevalence of HPV observed in the present study. Moreover, differences could be due to geographical location, ethnicity, sexual behavior, and the techniques used for HPV detection, among other factors. In any case, it is necessary to consider that the detection of HPV infection in young women is mainly at the expense of transient infections [39]. The relative increase in the prevalence of HPV detected in indigenous women with a median age of 30 years may be more likely to be associated with persistent infections, which could be a risk factor for the development of CC in this population.

In our study, the prevalence of HPV and HR HPV was higher in women under 20 years of age compared to those who were older. This coincides with other studies worldwide, which also show a high peak in the group of women between 15 and 25 years old [34,40]. It is estimated that seven out of ten sexually active women are infected with HPV at least once in their lifetime, and 74% of new infections occur between the ages of 15 and 24 [41]. The fact that the highest prevalence rates are found among adolescents and young adults makes them a high-risk group for developing premalignant and malignant lesions of the cervix [42].

It is imperative to note that the prevalence of HR HPV is uniform worldwide, with HPV16 being the most prevalent HR HPV type among women with normal cytology across all regions. Nevertheless, variations are apparent in the second most prevalent type among global regions. For instance, in America and Oceania, HPV51 is the second most common type, whereas, in Europe, it is HPV31; in Asia, it is HPV52; and in Africa, it is HPV58 [43]. Previously published studies of Ecuadorian women from different ethnicities demonstrated that the most prevalent HR HPV genotypes are 16, 18, 31, 39, 52, 53, 58 and 59 [44]. The genotypes most frequently found in this study were 58 and 59 (29.4%), 39 (3.79%), 42 (2.78%), 16 and 31 (2.53%), 66, 68 and 54 (2.27%); less frequent genotype 18 (0.76%) was detected. These results are inconsistent with data from other studies since for genotype 16, prevalence ranges of 13.1%, and for genotype 18, 2.7% have been reported [25,45]. These discrepancies could be explained by the conditions and use of different diagnostic methods.

In this study, a very high frequency of STIs (79.29%, CI 75.3–83.28) was detected, with 70.71% having more than one sexually transmitted pathogen. Although 50% of women reported a single sexual partner, their male partners might not be monogamous or had several other lifetime sexual partners, which may explain an increased risk of acquiring an STI. These findings are proportionally higher than the ones reported by Abad et al., where the frequency of STIs was 49.02% [46]. This difference could be explained by the association between some sexually transmitted pathogens and ethnicity.

A significant association was found in this study between HPV and Chlamydia trachomatis (OR 2.54, CI 1.08–5.99), which is similar to the findings of Ancer [47] and Ntuli [38]. It may be important to assess infected women with HPV co-infection with C. trachomatis, considering that this association may increase the risk of developing cervical cancer as a result of an inflammatory response [17]. However, the mechanism by which these co-infections favor the development of CC has not been elucidated.

## 5. Conclusions

Although there have been previous epidemiological studies on the prevalence of HPV in indigenous populations from several South American countries, none of them have studied the aforementioned communities in the southern region of Ecuador.

A high frequency of HPV and other STIs was observed in the studied population of indigenous women in Ecuador. In addition, this work suggests that HPV diagnosis is associated with C. trachomatis infection. Being younger than 20 years of age, having more than three sexual partners, and the presence of M. hominis was also associated with an increased risk of HPV infection. The highest-risk genotypes that were most frequently found in this study were 58, 59, 39, 16, 31, 35, and 68.

These data confirm that the detection of genital infections may be important to reveal the simultaneous presence of different STIs in indigenous women with low-educational levels, difficulties in access to health services, and exposure to risk factors for infection by the HPV that is the cause of CC.

In the local academic context, this study helped to implement new molecular techniques for the detection of these infections in the laboratory of the University of Cuenca, Ecuador, that will be available for use by the national health system.

We suggest the development of new research that will allow us to improve knowledge of HPV genotypes in larger samples of indigenous populations in general and particularly in women with cervical lesions, to produce vaccines appropriate to the genotypes found. Based on the results, it will be possible to adopt measures for the treatment, monitoring, and prevention of these infections, as well as the promotion of sexual and reproductive health, from a culturally appropriate perspective.

## Figures and Tables

**Table 1:** Frequency of genital infection by genotypes of HPV in indigenous women of the communities of Quilloac, Sevilla Don Bosco and Saraguro.

Genotype		Kañari			Shuar		Saraguro			Total		
Subtype	*n* = 131	%	CI 95%	*n* = 145	%	CI 95%	*n* = 120	%	CI 95%	*n* = 396	%	CI 95%
16 *	0	(0.00)	-	5	(3.45)	0.48–6.42	5	(4.17)	0.59–7.74	10	(2.53)	0.98–4.07
18 *	1	(0.76)	0.00–2.25	1	(0.69)	0.00–2.04	1	(0.83)	0.00–2.46	3	(0.76)	0.00–1.61
26	0	(0.00)	-	0	(0.00)	-	2	(1.67)	0.00–3.96	2	(0.51)	0.00–1.2
31 *	2	(1.53)	0.00–3.63	6	(4.14)	0.9–7.38	2	(1.67)	0.00–3.96	10	(2.53)	0.98–4.07
33 *	0	(0.00)	-	1	(0.69)	0.00–2.04	0	(0.00)	-	1	(0.25)	0.00–0.75
35 *	0	(0.00)	-	5	(3.45)	0.48–6.42	5	(4.17)	0.59–7.74	10	(2.53)	0.98–4.07
39 *	2	(1.53)	0.00–3.63	7	(4.83)	1.34–8.32	6	(5.00)	1.1–8.9	15	(3.79)	1.91–5.67
45 *	0	(0.00)	-	3	(2.07)	0.00–4.39	0	(0.00)	-	3	(0.76)	0.00–1.61
51 *	1	(0.76)	0.00–2.25	3	(2.07)	0.00–4.39	1	(0.83)	0.00–2.46	5	(1.26)	0.16–2.36
52 *	1	(0.76)	0.00–2.25	4	(2.76)	0.09–5.42	1	(0.83)	0.00–2.46	6	(1.52)	0.31–2.72
53 *	3	(2.29)	0.00–4.85	5	(3.45)	0.48–6.42	0	(0.00)	-	8	(2.02)	0.63–3.41
56 *	0	(0.00)	-	1	(0.69)	0.00–2.04	1	(0.83)	0.00–2.46	2	(0.51)	0.00–1.2
58 *	9	(6.87)	2.54–11.2	7	(4.83)	1.34–8.32	1	(0.83)	0.00–2.46	17	(4.29)	2.3–6.29
59 *	4	(3.05)	0.11–6.00	9	(6.21)	2.28–10.13	4	(3.33)	0.12–6.55	17	(4.29)	2.3–6.29
66 *	2	(1.53)	0.00–3.63	4	(2.76)	0.09–5.42	3	(2.50)	0.00–5.29	9	(2.27)	0.8–3.74
68 *	0	(0.00)	-	5	(3.45)	0.48–6.42	5	(4.17)	0.59–7.74	10	(2.53)	0.98–4.07
69	1	(0.76)	0.00–2.25	2	(1.38)	0.00–3.28	0	(0.00)	-	3	(0.76)	0.00–1.61
73	0	(0.00)	-	0	(0.00)	-	0	(0.00)	-	0	(0.00)	-
82	1	(0.76)	0.00–2.25	1	(0.69)	0.00–2.04	3	(2.50)	0.00–5.29	5	(1.26)	0.16–2.36
6	3	(2.29)	0.00–4.85	2	(1.38)	0.00–3.28	1	(0.83)	0.00–2.46	6	(1.52)	0.31–2.72
11	0	(0.00)	-	2	(1.38)	0.00–3.28	0	(0.00)	-	2	(0.51)	0.00–1.2
40	0	(0.00)	-	1	(0.69)	0.00–2.04	0	(0.00)	-	1	(0.25)	0.00–0.75
42	1	(0.76)	0.00–2.25	9	(6.21)	2.28–10.13	1	(0.83)	0.00–2.46	11	(2.78)	1.16–4.4
43	2	(1.53)	0.00–3.63	0	(0.00)	-	2	(1.67)	0.00–3.96	4	(1.01)	0.03–1.99
44	0	(0.00)	-	1	(0.69)	0.00–2.04	1	(0.83)	0.00–2.46	2	(0.51)	0.00–1.2
54	2	(1.53)	0.00–3.63	4	(2.76)	0.09–5.42	3	(2.50)	0.00–5.29	9	(2.27)	0.8–3.74
61	0	(0.00)	-	4	(2.76)	0.09–5.42	2	(1.67)	0.00–3.96	6	(1.52)	0.31–2.72
70	2	(1.53)	0.00–3.63	3	(2.07)	0.00–4.39	0	(0.00)	-	5	(1.26)	0.16–2.36

*n* = sample size. CI 95%: Confidence interval. * HPV HR.

**Table 2:** Frequency of genital infection by genotypes of HPV, HR HPV and LR HPV in indigenous women of the communities of Quilloac, Sevilla Don Bosco and Saraguro.

	Kañari			Shuar			Saraguro			Total		
Genotype	*n* = 131	%	CI 95%	*n* = 145	%	CI 95%	*n* = 120	%	CI 95%	*n* = 396	%	CI 95%
Any type HPV	25	(19.08)	12.35–25.81	58	(40.00)	32.03–47.97	29	(24.17)	16.51–31.83	112	(28.28)	23.85–32.72
HR HPV	19	(14.50)	8.47–20.53	47	(32.41)	24.8–40.03	27	(22.50)	15.03–29.97	93	(23.48)	19.31–27.66
LR HPV	9	(6.87)	2.54–11.2	24	(16.55)	10.5–22.6	8	(6.67)	2.2–11.13	41	(10.35)	7.35–13.35
2 or more types of HPV	9	(6.87)	2.54–11.2	18	(12.41)	7.05–17.87	10	(8.33)	3.39–13.28	37	(9.34)	6.48–12.21

*n* = sample size. CI 95%: Confidence interval. Any type of human papilloma virus HPV. HR HPV: High-risk human papillomavirus. LR HPV: Low-risk human papillomavirus.

**Table 3:** Association between cytological findings and HR HPV in indigenous women of the communities of Quilloac, Sevilla Don Bosco and Saraguro.

	HR HPV				
Lesion	*n* = 93	%	OR	CI 95%	
ASC-US	16	17.20	4.29	2.01	9.17
AGC-NOS	1	1.08	1.64	0.15	18.25
LSIL	10	10.75	5.96	2.11	16.89
HSIL	5	5.38	17.16	1.98	148.81
Any Intraepithelial lesion	30	32.26	5.80	3.16	10.65
Inflammation	90	96.77	4.69	1.42	15.53

ASC-US: Atypical Squamous Cells of Undetermined Significance. AGC-NOS: Atypical Glandular Cells, Not Otherwise Specified. LSIL: Low-grade Squamous Intraepithelial Lesion. HSIL: High-grade Squamous Intraepithelial Lesion. OR: odds ratio. CI 95%: Confidence interval.

**Table 4:** Frequency of infection by sexually transmitted pathogens according to age groups, in indigenous women of the communities of Quilloac, Sevilla Don Bosco and Saraguro.

Pathogens STI	≤19 Years			20–39 Years			40 Years and Over			Total		
	*n* = 33	(%)	CI 95%	*n* = 293	(%)	CI 95%	*n* = 70	(%)	CI 95%	*n* = 396	(%)	CI 95%
C trachomatis	4	(12.12)	0.99–23.26	19	(6.48)	3.66–9.3	1	(1.43)	0.00–4.21	24	(6.06)	3.71–8.41
N gonorrhoeae	1	(3.03)	0.00–8.88	0	(0.00)	-	1	(1.43)	0.00–4.21	2	(0.51)	0.00–1.2
T vaginalis	0	(0.00)	-	13	(4.44)	2.08–6.79	2	(2.86)	0.00–6.76	15	(3.79)	1.91–5.67
M hominis	17	(51.52)	34.46–68.57	83	(28.33)	23.17–33.49	13	(18.57)	9.46–27.68	113	(28.54)	24.09–32.98
M genitalium	0	(0.00)	-	4	(1.37)	0.04–2.69	1	(1.43)	0.00–4.21	5	(1.26)	0.16–2.36
U urealyticum	6	(18.18)	5.02–31.34	66	(22.53)	17.74–27.31	14	(20.00)	10.63–29.37	86	(21.72)	17.66–25.78
U parvum	26	(78.79)	64.84–92.74	170	(58.02)	52.37–63.67	32	(45.71)	34.04–57.38	228	(57.58)	52.71–62.44
STI	31	(93.94)	85.8–100.00	238	(81.23)	76.76–85.7	45	(64.29)	53.06–75.51	314	(79.29)	75.3–83.28
HPV	15	(45.45)	28.47–62.44	85	(29.01)	23.81–34.21	12	(17.14)	8.31–25.97	112	(28.28)	23.85–32.72
HR HPV	13	(39.39)	22.72–56.07	70	(23.89)	19.01–28.77	10	(14.29)	6.09–22.48	93	(23.48)	19.31–27.66
LR HPV	5	(15.15)	2.92–27.38	32	(10.92)	7.35–14.49	4	(5.71)	0.28–11.15	41	(10.35)	7.35–13.35

*n* = sample size. CI 95%: Confidence interval. STI: Any sexually transmitted pathogens CT, NG, TV, MH, MG, UU, UP. HPV: Any type of human papilloma virus. HR HPV: High-risk human papillomavirus. LR HPV: Low-risk human papillomavirus.

**Table 5:** Bivariate analysis of factors associated with human papillomavirus infection in indigenous women.

Variables	With HPV		Without HPV		OR	CI 95%	With HR HPV		Without HR HPV		OR	CI 95%
	*n* = 112	(%)	*n* = 284	(%)			*n* = 93	(%)	*n* = 303	(%)		
Age ≤ 19 years	15	(13.39)	18	(6.34)	2.29	1.11–4.71	13	14.00	20	6.60	2.30	1.09–4.82
Low socio-economic stratum	52	46.43	129	45.42	1.04	0.67–1.61	41	44.10	140	46.20	0.91	0.57–1.46
Single civil status	26	(23.21)	45	(15.85)	1.61	0.93–2.76	24	25.80	47	15.50	1.89	1.08–3.13
First sexual relation ≤ 19 years	99	(88.39)	247	(86.97)	1.14	0.58–2.24	85	91.40	261	86.10	1.70	0.77–3.78
≥1 abortion	37	(33.04)	65	(22.89)	1.66	1.03–2.69	29	31.20	73	24.10	1.42	0.85–2.38
>3 sexual partners	20	(17.86)	28	(9.86)	2.03	1.09–3.78	17	18.50	31	10.30	1.98	1.04–3.77
Oral contraceptives	26	(23.21)	66	(23.24)	1.00	0.59–1.68	22	23.70	70	23.10	1.03	0.59–1.78
Previous family history of CC	14	(12.50)	16	(5.63)	2.39	1.13–5.08	10	10.80	20	6.60	1.70	0.76–3.78
Chlamydia trachomatis	14	(12.50)	10	(3.52)	3.91	1.68–9.1	11	11.80	13	4.30	2.99	1.29–6.92
Mycoplasma hominis	43	(38.39)	70	(24.65)	1.91	1.19–3.04	36	38.70	77	25.40	1.85	1.13–3.02
Mycoplasma genitalium	2	(1.79)	3	(1.06)	1.70	0.28–10.33	2	2.20	3	1.00	2.19	0.36–13.35
Ureaplasma urealyticum	29	(25.89)	57	(20.07)	1.39	0.83–2.32	24	25.80	62	20.50	1.35	0.78–2.32
Ureaplasma parvum	74	(66.07)	154	(54.23)	1.64	1.04–2.59	60	64.50	168	55.40	1.46	0.90–2.36
Inflammation	109	(97.32)	262	(92.25)	3.05	0.89–10.4	90	96.80	281	92.70	2.34	0.69–8.03

*n* = sample size. OR: odds ratio. CI 95%: Confidence interval. CC: cervical cancer.

**Table 6:** Logistic regression analysis of factors associated with high-risk oncogenic human papillomavirus infection in indigenous women.

Variables	B	S.E	Wald	Df	*p*	OR	CI 95%
Age ≤ 19 years	0.740	0.390	3.604	1	0.058	2.09	0.98–4.49
>3 sexual partners	0.688	0.337	4.177	1	0.041	1.99	1.03–3.85
Chlamydia trachomatis	0.934	0.437	4.568	1	0.033	2.54	1.08–5.99
Mycoplasma hominis	0.482	0.260	3.445	1	0.063	1.62	0.97–2.69

B: Beta Coefficient. S.E: Standard Error. Wald: Wald’s Chi-square test. Df: Degrees of freedom. *p*: *p*-value. OR: Odds ratio. CI: Confidence interval.

## Data Availability

Not applicable.
